# Research on Unit Availability Assessment Model for Roadway High-Entropy Energy Integrating Output Capacity and Behavioral Orderliness

**DOI:** 10.3390/e28070748

**Published:** 2026-07-01

**Authors:** Juexiao Chen, Yinlin He, Lei Shi, Yihao Tao, Donghuan Liu

**Affiliations:** 1 College of Automotive and Energy Engineering, Tongji University, Shanghai 201804, China; chenjuexiao@tongji.edu.cn (J.C.); 2011681@tongji.edu.cn (L.S.); h5jhkjkk@163.com (Y.T.); 2 CRRC Qingdao Sifang Co., Ltd., Qingdao 266111, China; liudonghuan_sf@163.com

**Keywords:** roadway high-entropy energy, unit availability, output capacity, behavioral orderliness, fuzzy entropy

## Abstract

Due to the stochastic and fluctuating nature of output from roadway high-entropy energy units, traditional evaluation metrics based on fault statistics struggle to comprehensively assess their availability. To address this, this paper proposes a novel unit availability assessment method that integrates output capacity and behavioral orderliness, defining availability as the mathematical product of an output capacity factor and a behavioral orderliness factor. By overcoming the intrinsic flaws of conventional binary status judgments, this approach yields intuitive and logical evaluation results upon case study validation. The proposed model successfully distinguishes the comprehensive performance of equipment under diverse output conditions, thereby offering a fresh perspective for the refined evaluation of roadway high-entropy energy units.

## 1. Introduction

The profound integration of transportation and energy systems has emerged as a crucial strategic pathway for achieving the “Dual Carbon” goals [1,2]. The large-scale deployment of intelligent connected technologies, roadside sensing equipment, and electric vehicle charging infrastructure has generated massive distributed energy loads along highways, which imposes more stringent demands on the reliability and continuity of power supply [3]. In this context, exploiting and utilizing roadway high-entropy energy introduces a novel solution for self-consistent highway energy systems [4,5]. Research indicates that deploying equipment capable of harvesting ambient energy—such as wind, solar, and traffic-induced vibration—along highways can facilitate on-site power supply for traffic loads at service areas, tunnels, and bridges, consequently enhancing the energy self-sufficiency rate [6,7].

In this study, the term “roadway high-entropy energy” is used as an operational engineering descriptor rather than as a thermodynamic or materials-science concept. It denotes distributed stochastic roadside energy resources deployed along transport infrastructure, whose outputs are spatially dispersed, temporally variable, and coupled with traffic and meteorological uncertainty. Here, “high-entropy” refers to the high uncertainty of the roadside energy-output process and does not imply a thermodynamic entropy state or a high-entropy material composition. This operational usage is also distinct from the fuzzy entropy introduced later, which is used only as a quantitative time-series complexity metric.

Unlike traditional centralized energy generation, roadway high-entropy energy systems exhibit prominent spatiotemporal randomness and output volatility [8]. This characteristic poses severe challenges to the operational evaluation of these systems. Existing evaluation methods for energy systems have primarily evolved along two distinct trajectories: one focuses on the reliability assessment of power systems, emphasizing equipment faults and outage probabilities with typical indices like power supply reliability and loss of load frequency [9,10]; the other centers on analyzing the output characteristics of renewable energy, focusing on generation potential, such as theoretical and technical potentials [11,12]. Recently, scholars have attempted to merge these two paradigms. Trivedi et al. systematically summarized reliability and availability assessment methods based on stochastic models, highlighting the application value of state-space models during the system design phase [13]. Ning et al. developed a supply-demand matching evaluation framework for self-consistent highway energy systems, encompassing ten indicators like self-sufficiency rate and supply reliability, while employing sequential Monte Carlo methods to quantify source-load uncertainties [6]. Furthermore, Shen et al. explored cascading failures and recovery mechanisms of power supply systems for intelligent connected roads under extreme events from a resilience perspective [3].

Nevertheless, the aforementioned studies still present several deficiencies. First, traditional reliability indicators predominantly target failure events and thus struggle to capture the continuous fluctuation traits of renewable energy outputs [14]. Second, analyses of output characteristics mostly remain at the resource assessment level without effectively bridging to equipment operational status evaluations [15]. Intrinsically, the volatility of equipment output reflects the degree of order in its operational state, and entropy naturally serves as a robust tool to quantify this orderliness. Entropy theories have already been applied in energy system evaluations; for instance, entropy generation analysis is utilized to uncover sources of irreversible losses in electrochemical systems [16], while the entropy weight method provides objective weighting in the comprehensive evaluation of coal-fired units [17]. However, systematic research on employing entropy theory to quantify the behavioral orderliness of equipment output—and subsequently integrating it with output capacity to formulate an availability index—remains absent.

To address these gaps, this paper proposes a unit availability assessment method for roadway high-entropy energy that amalgamates output capacity and behavioral orderliness. The core concept lies in decomposing unit equipment availability into two dimensions: “output capacity” and “behavioral orderliness”. The former reflects the energy production level of the equipment, while the latter portrays the stability and certainty of its output behavior. The combination of these two elements enables a quantitative characterization of the equipment’s comprehensive performance.

The remainder of this paper is organized as follows: Section 2 constructs the unit availability assessment model, detailing the calculation methodologies for both the output capacity factor and the behavioral orderliness factor. Section 3 conducts a case study validation based on German wind power data, analyzing the availability calculation results of four typical virtual devices. Section 4 summarizes the entire paper and suggests directions for future research.

## 2. Unit Availability Assessment Model

The unit availability assessment model proposed in this paper relies on the following assumptions:

1. The power time series of the unit equipment is completely observable, and the data sampling intervals are uniform.
2. The unit equipment operates continuously during the evaluation period, disregarding prolonged shutdowns caused by maintenance or faults.
3. The external resources surrounding the unit equipment do not undergo structural changes within the evaluation period, meaning output fluctuations primarily stem from natural randomness rather than long-term variations in resource endowment.
4. The output behaviors of individual unit equipment are mutually independent, and the impact of spatiotemporal correlations among equipment on availability is temporarily excluded.

This model is suitable for evaluating the availability of renewable energy generation units within roadway high-entropy energy systems, particularly in scenarios necessitating a comprehensive consideration of energy production efficiency and operational stability. For equipment with special operational modes, such as frequent start-stops or rapid power regulation, adjustments to parameters or the model itself must be made in accordance with actual conditions.

### 2.1. Definition and Composition of Unit Availability

In traditional power system reliability engineering, unit availability is conventionally defined as the probability that an equipment is in a functional state at any given moment, calculated via random processes of failure and repair [9]. However, for generation units in roadway high-entropy energy systems, their operation is not a simple dichotomy of “normal” and “faulty” states. Even in a fault-free state, the equipment’s output might remain exceedingly low due to insufficient excitation, resulting in a highly limited actual contribution to the system. Consequently, indices relying solely on fault statistics are inadequate for comprehensively delineating the availability of roadway high-entropy energy equipment.

To overcome this limitation, this study redefines unit availability through the dual dimensions of energy production and operational quality. On the one hand, the value of unit equipment is initially manifested in its power generation capacity; equipment with a higher average output level contributes more significantly to the system. On the other hand, the smoothness and certainty of the equipment’s output directly influence the system’s dispatchability and grid-friendliness, as highly volatile equipment amplifies system reserve costs and regulatory pressures. Therefore, this paper defines unit availability $A_{unit}$ as the product of the output capacity factor and the behavioral orderliness factor

(1) $$A_{unit}=C_{cap}\cdot F\left(E\right)$$

where $C_{cap}$ represents the output capacity factor, reflecting the average output level of the equipment during the evaluation period, with values ranging from [0,1]. $F\left(E\right)$ denotes the behavioral orderliness factor, describing the smoothness and certainty of the equipment’s output time series, with a value range of (0,1]. Equation (1) indicates that equipment can achieve high availability only when it concurrently exhibits a robust output level and a stable, orderly operational state.

### 2.2. Model of Output Capacity Factor

The output capacity factor signifies the average energy production level of the unit equipment over the evaluation period. Assume the evaluation period comprises T equally spaced sampling points (for instance, using hourly data over a one-year period yields T=24×365=8760. Let the normalized power time series of the equipment be $p\left(t\right)\in \left[0,1\right],t=1,2,\ldots ,T$, where each point in the series is an output value normalized against the unit’s installed capacity or reference power. The output capacity factor is defined as the arithmetic mean of this series:

For heterogeneous equipment, the default denominator for unit-level availability should be each unit’s own installed or rated capacity, namely its maximum physically admissible output. This own-rated normalization measures capacity-utilization efficiency and avoids penalizing smaller units solely because of their nominal size. A common reference capacity, such as the largest rated capacity in the comparison set, can be reported as a complementary size-weighted contribution indicator; in that case, the common-reference capacity factor satisfies $C_{i}^{ref}=C_{i}\frac{P_{i}^{rated}}{P_{ref}}$. Thus, the two normalizations answer different questions and should be interpreted separately.

(2) $$C_{cap}=\frac{1}{T}\sum_{i=1}^{T}p(t)$$

$C_{cap}$ measures the equipment’s capacity utilization degree during the assessment cycle, representing the ratio of actual energy generated to the potential maximum generation. A $C_{cap}$ approaching 1 implies sustained high-level output throughout the period, whereas a lower value indicates prolonged low-output states.

Notably, $C_{cap}$ relies exclusively on the mean of the power series and remains insensitive to internal fluctuations. Consequently, two units with identical average outputs will attain the same $C_{cap}$ value, regardless of whether their actual generation is stable or highly erratic. This inherent insensitivity underscores the necessity of introducing the behavioral orderliness factor.

### 2.3. Behavioral Orderliness Factor Based on Fuzzy Entropy

#### 2.3.1. Definition and Rationale for Introducing Fuzzy Entropy

Entropy serves as a classical metric for quantifying the disorder or uncertainty of a system. In time series analysis, methods like approximate entropy and sample entropy have been broadly deployed to gauge complexity in fields such as physiological signals and mechanical vibrations [18]. Nevertheless, the traditional definition of these entropies employs a binary similarity criterion: distances between two vectors falling below a threshold are deemed similar, and those above are not. This binary approach entails two major issues: first, minuscule variations near the threshold can trigger abrupt changes in entropy values, leading to a lack of continuity; second, it exhibits heightened sensitivity to noise and poor stability with small sample sizes [19].

To resolve these issues, Chen et al. [20] introduced the concept of fuzzy entropy in 2007, incorporating fuzzy set theory into entropy measurement. The fundamental premise involves substituting binary similarity judgments with a continuous exponential fuzzy membership function, mapping the Chebyshev distance between vectors into a continuous similarity within the [0, 1] interval. This innovation yields enhanced relative consistency, noise resistance, and parameter continuity.

#### 2.3.2. Calculation Steps for Fuzzy Entropy

For an equipment’s normalized power time series {p(t),t=1,2,…,T} of length T, the fuzzy entropy calculation steps are as follows [19,20]:

1. Phase Space Reconstruction

Define an embedding dimension m and construct T−m+1 vectors of m dimensions: $X_{i}^{m}=\left\{p\left(i\right),p\left(i+1\right),\ldots ,p\left(i+m-1\right)\right\},i=1,2,\ldots ,T-m+1$.

2. Define Inter-Vector Distance

For i=1,2,…,T−m+1 and j=1,2,…,T−m+1, with i≠j, the Chebyshev distance between vectors $X_{i}^{m}$ and $X_{j}^{m}$ is defined as

(3) $$d_{ij}^{m}=\underset{k=0,1,\ldots ,m-1}{\max}\left|X_{i}^{m}(k)-X_{j}^{m}(k)\right|$$

where $X_{i}^{m}(k)$ is the k-th element of vector $X_{i}^{m}$, k=0,1,…,m−1, and k=0 corresponds to the first element.

3. Calculate Similarity of Vector Pairs

The similarity of vector $X_{j}^{m}$ to $X_{i}^{m}$ (i≠j) is computed using an exponential fuzzy function

(4) $$\mu_{ij}^{m}=exp(-{\left(\frac{d_{ij}^{m}}{r}\right)}^{n})$$

In Equation (4), r denotes the similarity tolerance that dictates the width of the exponential function, while n represents the fuzzy power exponent controlling the gradient of similarity attenuation with distance.

4. Calculate Average Similarity

For each i=1,2…,T−m+1, calculate the mean similarity with all i≠j vectors

(5) $$\Phi_{i}^{m}(r)=\frac{1}{T-m}\sum_{j=1,j\neq i}^{T-m+1}\mu_{ij}^{m}$$

Subsequently, average across all i to derive the mean similarity in the m-dimensional space

(6) $$\Phi^{m}(r)=\frac{1}{T-m+1}\sum_{i=1}^{T-m+1}\Phi_{i}^{m}(r)$$

5. Calculate Average Similarity in m+1 Dimensions

Expand the embedding dimension to m+1 and iterate steps 1. through 4. to acquire the mean similarity $\Phi^{m+1}(r)$. At this stage, the number of constructed vectors becomes T−m.

6. Calculate Fuzzy Entropy

Fuzzy entropy E is defined as

(7) $$E(m,r,n)=\ln\Phi^{m}(r)-\ln\Phi^{m+1}(r)$$

Physically, fuzzy entropy quantifies the probability of generating new patterns as the time series dimensionality alters. A higher entropy value indicates diminished self-similarity and escalated complexity, reflecting profound randomness and volatility in the series. Conversely, a lower entropy value suggests a more regular and stable sequence.

#### 2.3.3. Principles for Parameter Selection

The computation of fuzzy entropy depends on three pivotal parameters: embedding dimension m, similarity tolerance r, and fuzzy power exponent n. The selection of these parameters directly influences the discriminatory capacity and stability of entropy values, governed by the following principles [20]:

1. Embedding dimension m: This parameter dictates the length of the reconstructed vectors. An excessively large m truncates the effective data length, decreasing the number of comparable vector pairs and thereby degrading statistical stability; an unduly small m may fail to capture the dynamic information of the sequence. In relevant entropy calculation literature [20,21,22], m=2 serves as the most prevalent default, striking an optimal balance between data length and information preservation. Consequently, this paper adopts m=2 as the baseline for the embedding dimension.
2. Similarity tolerance r: This parameter governs the width of the exponential function. A larger r induces a slower attenuation of similarity relative to distance, mitigating the impact of noise-induced distance perturbations on similarity, yet it simultaneously blunts sensitivity to subtle sequence variations. Conversely, a smaller r enforces stricter similarity criteria but amplifies noise sensitivity. Typically, r is assigned a value equivalent to 0.1 to 0.25 times the standard deviation σ of the equipment’s normalized power series [20]. This paper establishes r=0.2σ as the standard value. Because a tolerance proportional to each device’s own standard deviation may affect cross-device comparability, the present analysis distinguishes two uses of r: a local tolerance, $r_{i}=0.2\sigma_{i}$, for characterizing the intrinsic complexity of an individual sequence, and a fixed reference tolerance, $r_{0}=0.2\sigma_{ref}$, for robustness checks across devices. In the case study, replacing the local tolerance with the fixed reference tolerance preserved the pairwise distinction between stable and fluctuating devices, indicating that the ranking is not driven by the tolerance definition alone.
3. Fuzzy power exponent n: This metric controls the gradient of similarity decay over distance. When n=2, the membership function adopts a Gaussian profile where the decay gradient near $d_{ij}^{m}=0$ approaches zero. This characteristic ensures that minor short-range perturbations exert negligible influence on similarity, thereby fortifying the method’s noise immunity. Consistent with the default setting established by Chen et al. [20] upon proposing fuzzy entropy, this study adopts n=2.

#### 2.3.4. Definition of the Behavioral Orderliness Factor

Leveraging the properties of fuzzy entropy described above, this paper delineates the behavioral orderliness factor F(E) as the negative exponential mapping of the fuzzy entropy of the equipment’s power series

(8) F(E)=exp(−λE)

Here, E denotes the calculated fuzzy entropy of the target equipment’s power sequence, and λ (λ>0) functions as a scale adjustment parameter controlling the impact of the entropy value on orderliness. This specific mapping possesses several distinct attributes:

The negative exponential mapping was selected because it is monotonic, strictly positive, equals one at zero entropy, and is analogous to reliability attenuation functions in which increasing disorder continuously reduces an effective performance factor. It also avoids the abrupt cutoff that can occur in linear mappings under high-entropy conditions. Nevertheless, the mapping form is a modeling choice; therefore, the case study includes a comparison with reciprocal and linear monotonic mappings to test whether the final ranking depends on this functional form.

1. Monotonicity: F(E) decreases monotonically with escalating E, meaning a greater entropy value correlates to diminished behavioral orderliness.
2. Closed Domain: For E∈[0,∞), it follows that F(E)∈(0,1].
3. Adjustable Parameters: By calibrating λ, one can dictate the sensitivity of behavioral orderliness relative to the entropy value. A larger λ results in a lower behavioral orderliness for an identical entropy value, reflecting stricter assessment criteria. The selection of scale parameter λ should be tailored to specific application scenarios. This paper employs λ=1 as the baseline in case studies, while the influence of λ on evaluation outcomes will be further scrutinized in subsequent discussions.

The introduction of the behavioral orderliness factor F(E) empowers the availability evaluation to effectively distinguish among equipment that share comparable output levels but manifest vastly disparate operational stabilities. Equipment operating stably exhibits robust power sequence regularity and small fuzzy entropy, culminating in a high F(E) value. In stark contrast, severely fluctuating equipment displays intense randomness and large fuzzy entropy, yielding a suppressed F(E) score.

### 2.4. Discussion on the Unit Availability Model

An analysis of the proposed unit availability model reveals the following characteristics:

1. Closed Value Range

Given that $C_{cap}\in [0,1]$ and F(E)∈(0,1], the resulting availability $A_{unit}\in [0,1]$, facilitating straightforward horizontal comparisons across different devices. When a unit generates no power, $C_{cap}=0$ and $A_{unit}=0$. Conversely, when a unit consistently maintains maximum output with perfect orderliness, $C_{cap}=1$ and F(E)=1, culminating in an ideal $A_{unit}=1$.

2. Explicit Physical Significance

The output capacity factor $C_{cap}$ embodies the energy yield efficiency of the unit, whereas the behavioral orderliness factor F(E) characterizes the operational smoothness and certainty. Their product encapsulates a holistic balance between the quantity and quality of the unit’s energy output. Highly available equipment is capable of not only generating substantial power but also delivering it steadily.

3. Independent Evaluability

F(E) relies exclusively on the negative exponential mapping of the unit’s intrinsic fuzzy entropy and remains immune to the integration or removal of other devices. This autonomy renders the model highly suitable for the absolute assessment of individual units, streamlining historical comparisons across diverse scenarios and timeframes.

4. Parameter Flexibility

The scale parameter λ within the behavioral orderliness factor can be modulated to align with practical prerequisites, thereby altering the rigor of the evaluation. A diminutive λ implies a more lenient assessment suited for environments with high tolerance for volatility. Conversely, a robust λ denotes strict criteria, ideal for scenarios demanding the filtration of exceptionally stable equipment.

5. Cohesion with Classical Entropy Methods

Fuzzy entropy inherits the complexity measurement capabilities of approximate and sample entropies. Simultaneously, by employing a continuous similarity function, it mitigates the flaws inherent in traditional techniques, establishing a more resilient and dependable quantification of behavioral orderliness.

## 3. Case Study Verification

To verify the efficacy and rationality of the proposed unit availability evaluation method, this section constructs four virtual devices with archetypal operational traits based on authentic wind power output data procured from the German Open Power System Data platform. Subsequently, the output capacity factor, fuzzy entropy, behavioral orderliness factor, and final unit availability are calculated for each device. An analysis of these results validates the model’s proficiency in differentiating devices characterized by disparate output signatures.

### 3.1. Data Acquisition and Preprocessing

The foundational data utilized in this study stems from datasets published by the Open Power System Data project, encompassing hourly actual generation records of German onshore wind power from 2015 to 2017 (measured in MW). The cleaned dataset for the year 2016 was selected as the baseline; however, to simplify computations while preserving sufficient sequence length, the initial 1000 h (approximately 42 days) were extracted for the case study analysis. Data preprocessing entailed the following steps:

The first 1000 h were used as a compact demonstration window to keep the calculation transparent while providing enough points for stable fuzzy-entropy estimation. To address seasonal representativeness, an additional robustness check was performed on the available 2016 record, which comprises 8712 valid hourly observations; the record was divided into natural monthly windows, and the same indicators were recalculated in each window. The annual monthly window mean ranking remained A>B>C>D, consistent with the 1000 h demonstration.

1. Missing Value Handling: The initial 1000 h segment used in the main demonstration contained no missing entries. In the full-year robustness check, only valid hourly wind-generation observations were retained, and no interpolation or imputation was applied.
2. Total Output Normalization: Maximum value normalization was applied to the raw output series $P_{total}(t)$ to derive the reference sequence $p_{total}(t)\in [0,1]$.

(9) $$p_{total}(t)=\frac{P_{total}(t)}{max(P_{total}(t))}$$

This baseline sequence effectively retains the stochasticity, intermittency, and volatility inherently characteristic of actual wind power output. Calculations establish the mean of this normalized reference sequence at 0.405.

### 3.2. Construction of Typical Equipment

To generate equipment power sequences exhibiting diverse output levels and fluctuation profiles, this study utilizes a modulation model to derive four virtual devices from the baseline sequence $p_{total}(t)$. The normalized power series $p_{i}(t)$ for device i is synthesized via the following equation:

(10) $$p_{i}(t)=\alpha_{i}\cdot p_{total}(t)+\beta_{i}\cdot \varpi (t)+\gamma_{i}$$

where $\alpha_{i}$ acts as the output scaling factor modulating the average output magnitude; $\beta_{i}$ is the fluctuation intensity factor governing the amplitude of volatility; $\gamma_{i}$ serves as a baseline offset for fine-tuning the output baseline. The term ϖ(t) signifies a fluctuation noise source, extracted by applying a high-pass filter to the baseline sequence to isolate its high-frequency components, thereby simulating random operational perturbations.

The synthesized devices are stylized benchmark sequences designed to isolate the effects of output level and fluctuation intensity; they are not intended to reproduce the complete electromechanical behavior of a specific turbine. The perturbation term is mean-centered before superposition, and the resulting sequence is clipped to the normalized interval [0,1] to avoid physically impossible negative or above-rated outputs. For engineering deployment, the same assessment framework should preferably be applied to measured unit-level data whenever such data are available.

**Figure 1:**
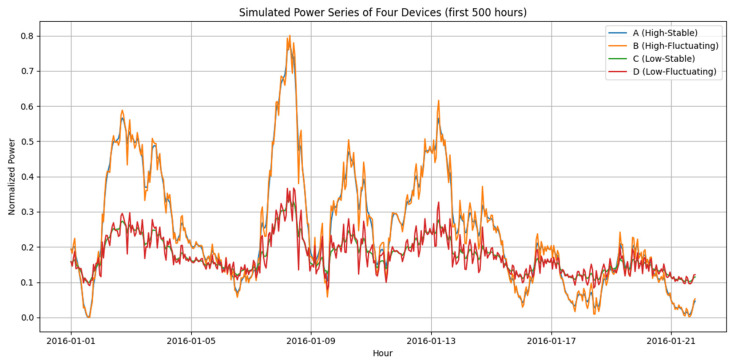
Comparison of normalized power series of four typical devices (first 500 h).

The parametric configurations for the four prototypical devices are detailed in Table 1:

Post-generation, values exceeding the [0, 1] interval were strictly truncated to preserve the validity of the design parameters. Figure 1 delineates the normalized power curves for the first 500 h of the four devices. A visual inspection confirms that the trajectory of Device A smoothly tracks the upper envelope of the total output. Device B, while sustaining high output, displays conspicuous high-frequency spikes. The overall output levels for Devices C and D lean lower; nevertheless, Device C maintains a relatively gradual curve, whereas Device D exhibits aggressive localized fluctuations. These observations affirm that the synthesized sequences successfully replicate the intended output characteristics.

### 3.3. Calculation Results and Analysis

Based on the unit availability model formulated in Section 2, the output capacity factor, fuzzy entropy, behavioral orderliness factor, and unit availability were sequentially computed for the four devices.

#### 3.3.1. Output Capacity Factor

The output capacity factors were computed according to Equation (2), yielding the results presented in Table 2.

Table 2 demonstrates that the mean outputs of Devices A and B are virtually equivalent, as are those of Devices C and D, corroborating the high and low output groupings established during parameter design. Concurrently, stable and fluctuating devices within the same cluster exhibit nearly identical output capacity factors, thereby validating $C_{cap}$’s inherent insensitivity to volatility.

#### 3.3.2. Fuzzy Entropy and Behavioral Orderliness Factor

Fuzzy entropy was evaluated using parameters m=2, n=2, and r=0.2σ (where σ is the standard deviation of each device’s specific sequence). The derived calculations are displayed in Table 3.

Insights drawn from Table 3 include:

- Stable devices (A and C) register markedly lower fuzzy entropy compared to their fluctuating counterparts (B and D), evidencing that their power series hold superior regularity and smoothness. Device C secures the minimal fuzzy entropy, while Device D logs the maximum, proving fuzzy entropy’s effectiveness in quantifying fluctuation severity.
- An inverse relationship exists between the behavioral orderliness factor and fuzzy entropy, leading to an F(E) ranking of C > A > B > D. This hierarchy accurately reflects the qualitative operational stability of the equipment, with C being the most stable and D the most erratic.

#### 3.3.3. Unit Availability

The final unit availability metrics are tabulated in Table 4.

Based on Table 4, the following conclusions emerge:

1. Devices with high output capabilities boast a substantially higher overall availability than low-output ones. Devices A and B dramatically outperform C and D, a phenomenon underscoring the dominant role of the output capacity factor.
2. When comparing devices with identical output levels, stable units consistently outperform fluctuating ones. The availability of Device A (high-output, stable) surpasses Device B (high-output, fluctuating) by roughly 8.4%; similarly, Device C (low-output, stable) edges out Device D (low-output, fluctuating) by approximately 16.8%. This substantiates that the behavioral orderliness factor effectively penalizes severe output volatility.
3. The proposed model commands robust comprehensive discriminative power, yielding an availability ranking of A > B > C > D. Although Device C exhibits supreme behavioral orderliness, its deficient output volume drags its composite availability below that of both A and B. This sequence harmonizes precisely with a balanced evaluation of quantity and quality.

### 3.4. Discussion

The scale parameter λ determines the behavioral orderliness factor’s sensitivity towards fuzzy entropy. To scrutinize the impact of λ on evaluation results, the behavioral orderliness factors for the four devices were recalculated utilizing λ=0.5, λ=1, and λ=2, yielding the outcomes in Table 5.

Table 5 reveals that regardless of the λ value applied, the F(E) ranking across the four devices persistently remains C > A > B > D. This consistency attests to the robustness of the evaluation results in relation to λ variations. As λ magnifies, F(E)’s sensitivity to entropy escalates, perpetually widening the gap between stable and fluctuating devices. For instance, the F(E) differential between Devices A and B expands from 0.039 at λ=0.5 to 0.140 at λ=2. When λ is small, the evaluative metric is more permissive, enabling fluctuating devices to still secure a relatively high orderliness score. Conversely, a larger λ applies stringent evaluation standards, causing a precipitous drop in the orderliness of fluctuating devices.

In practical scenarios, an appropriate λ should be chosen commensurate with the prevailing operational stability requirements. For rigorous screening targeting high stability, a larger λ is advisable; if the system demonstrates a high tolerance for fluctuations, a smaller λ suffices. This paper adopts λ=1 as a baseline to simultaneously accommodate sensitivity and universality.

As shown in Table 6, the ranking remains A>B>C>D under all three monotonic mappings. Therefore, the final availability ranking in this case is not an artifact of the negative exponential mapping.

To examine whether the case-study ranking depends on the normalization reference, a common-reference check was also considered. The four benchmark devices in this study were generated on the same normalized power base and therefore have an identical nominal reference capacity; consequently, $\frac{P_{i}^{rated}}{P_{ref}}=1$ and $C_{i}^{ref}=C_{i}$ for all four devices. The availability values in Table 4 therefore also represent the common-reference result, and the ranking remains A>B>C>D. For measured units with substantially different rated capacities, the common-reference result should be reported as a complementary system-contribution metric, while the own-rated result remains the unit-level availability metric. To further examine temporal robustness, Table 7 reports the monthly-window robustness check based on all valid 2016 wind-power observations, confirming that the availability ranking remains consistent with the demonstration-window results.

The full-year monthly window results confirm that high-output devices retain higher overall availability than low-output devices and that stable devices outperform their fluctuating counterparts within the same output group. This finding supports the use of the demonstration window for validating the model’s ranking behavior.

The case study outcomes confirm that the formulated unit availability evaluation method organically integrates the unit’s energy output efficiency with the orderliness of its operational state. The generated evaluation metrics are both intuitive and logically sound, adroitly differentiating the composite performance of equipment under multifaceted output conditions. Ultimately, this method lays a dependable foundational stratum for subsequent availability aggregation at the system and regional echelons.

## 4. Conclusions

Addressing the idiosyncratic operational characteristics of generation units embedded within roadway high-entropy energy systems, this paper introduces a unit availability evaluation methodology that amalgamates output capacity with behavioral orderliness. The principal contributions and conclusions are summarized as follows:

1. The traditional notion of unit availability is deconstructed into an output capacity factor and a behavioral orderliness factor. The former characterizes the average energy production capability of the equipment, while the latter employs fuzzy entropy to quantify the smoothness and deterministic nature of the output sequence. Together, they comprehensively portray the equipment’s operational performance across both quantitative and qualitative dimensions.
2. By integrating fuzzy entropy as the quantification instrument for behavioral orderliness and replacing conventional binary judgments with a continuous exponential similarity function, the method significantly augments noise robustness and stabilizes small-sample computations.
3. A case study was executed using authentic wind power data from Germany to synthesize four devices manifesting typical operational conditions. The empirical results demonstrate that the unit availability model proficiently balances output volume against operational stability, and the validation successfully affirms the model’s comprehensive assessment prowess for disparate output traits.

The unit availability evaluation method presented herein transcends the inherent limitations of traditional reliability metrics that fixate predominantly on failure events, thus equipping researchers with an innovative tool for the refined assessment of roadway high-entropy energy systems. Future investigative efforts will explore system-level availability aggregation methodologies by incorporating spatiotemporal correlations and complementarities among distinct devices. Moreover, actual operational data from a broader spectrum of equipment types will be integrated to further corroborate the method’s universal applicability.

Although the present case study uses synthesized benchmark devices to provide controlled comparisons, future work should extend the validation to measured device-level datasets across units with different installed capacities. This would allow the fixed-tolerance strategy and rated-capacity normalization to be further tested under real heterogeneous operating conditions.

## Figures and Tables

**Table 1:** Parameter configuration of typical devices.

Unit Number	Type	α	β	γ
A	High-Output Stable	1.0	0.05	0
B	High-Output Fluctuating	1.0	0.30	0
C	Low-Output Stable	0.3	0.05	0.1
D	Low-Output Fluctuating	0.3	0.30	0.1

**Table 2:** Calculation results of output capacity factor.

Device	Average Output	Output Capacity Factor
A	0.4050	0.405
B	0.4050	0.405
C	0.2215	0.222
D	0.2216	0.222

**Table 3:** Calculation results of fuzzy entropy and behavioral orderliness factor.

Device	Fuzzy Entropy	Behavioral Orderliness Factor
A	0.0423	0.959
B	0.1247	0.883
C	0.0286	0.972
D	0.1825	0.833

**Table 4:** Calculation results of unit availability.

Device	Output Capacity Factor	Behavioral Orderliness Factor	Unit Availability
A	0.405	0.959	0.388
B	0.405	0.883	0.358
C	0.222	0.972	0.216
D	0.222	0.833	0.185

**Table 5:** Behavioral orderliness factors under different scale parameters.

Device	Fuzzy Entropy	*λ* = 0.5	*λ* = 1	*λ* = 2
A	0.0423	0.979	0.959	0.919
B	0.1247	0.940	0.883	0.779
C	0.0286	0.986	0.972	0.944
D	0.1825	0.913	0.833	0.694

**Table 6:** Availability values under alternative monotonic mappings of fuzzy entropy.

Device	exp(−E)	$\frac{1}{1+E}$	1−E
A	0.388	0.389	0.388
B	0.358	0.360	0.354
C	0.216	0.216	0.216
D	0.185	0.188	0.181

**Table 7:** Monthly window robustness check using valid 2016 wind-power observations.

Device	Mean Capacity Factor	Mean Fuzzy Entropy	Mean Orderliness	Mean Availability
A	0.238	0.0575	0.944	0.225
B	0.238	0.0680	0.934	0.222
C	0.171	0.0287	0.972	0.166
D	0.171	0.0447	0.956	0.164

## Data Availability

The German Open Power System Data can be found and download at: https://open-power-system-data.org/.
